# P300 Event-Related Potential as an Indicator of Inattentional Deafness?

**DOI:** 10.1371/journal.pone.0118556

**Published:** 2015-02-25

**Authors:** Louise Giraudet, Marie-Eve St-Louis, Sébastien Scannella, Mickaël Causse

**Affiliations:** 1 DMIA, ISAE, Université de Toulouse, Toulouse, 31055, France; 2 École de Psychologie, Université Laval, Québec, Québec, G1V 0A6, Canada; Vanderbilt University, UNITED STATES

## Abstract

An analysis of airplane accidents reveals that pilots sometimes purely fail to react to critical auditory alerts. This inability of an auditory stimulus to reach consciousness has been coined under the term of inattentional deafness. Recent data from literature tends to show that tasks involving high cognitive load consume most of the attentional capacities, leaving little or none remaining for processing any unexpected information. In addition, there is a growing body of evidence for a shared attentional capacity between vision and hearing. In this context, the abundant information in modern cockpits is likely to produce inattentional deafness. We investigated this hypothesis by combining electroencephalographic (EEG) measurements with an ecological aviation task performed under contextual variation of the cognitive load (high or low), including an alarm detection task. Two different audio tones were played: standard tones and deviant tones. Participants were instructed to ignore standard tones and to report deviant tones using a response pad. More than 31% of the deviant tones were not detected in the high load condition. Analysis of the EEG measurements showed a drastic diminution of the auditory P300 amplitude concomitant with this behavioral effect, whereas the N100 component was not affected. We suggest that these behavioral and electrophysiological results provide new insights on explaining the trend of pilots’ failure to react to critical auditory information. Relevant applications concern prevention of alarms omission, mental workload measurements and enhanced warning designs.

## Introduction

In aeronautics, auditory alerts are known to present various advantages in emergency situations as compared to visual stimuli. They provide information for pilots without requiring head/gaze movements [1] and provoke faster reaction times [2]. Yet, the analysis of air safety reports reveals that a significant number of accidents are due to a lack of reaction or misperception to auditory alarms [3]. Two reasons related to cognitive biases are usually proposed to account for such a lack of response. The primary explanation is the ‘cry-wolf effect’ [4] resulting from a high false-positive rate from the alerting system which leads to pilots mistrusting alarms [5–7], especially under high workload conditions [8]. The second explanation is that the aggressive, distracting, and disturbing nature of auditory alarms [1,9] can considerably increase pilot stress levels during warning events [10]. In fact, the immediate reaction for many pilots is to find a way to consciously silence the noise, rather than analyze the meaning of the alert. Nevertheless, these reasons are not sufficient to fully explain the misperception of critical auditory warnings as often reported in accident analyses [11] and observed in flight simulators [12]. A complementary explanation is the role of the sustained mental processes engaged in the cockpit. Despite the impressive complexity and processing power of the human brain, it exhibits severe capacity limits in information processing; the neural basis of which has been shown in neuroimaging studies [13,14]. In addition, goal-directed behavior requires focusing attention on goal-relevant stimuli while ignoring irrelevant distractors. The occurrence of an alarm during a nominal period of piloting is often unexpected for the crew and unrelated to their current task.

Moreover, there is evidence that tasks involving a high perceptual load consume most of the attentional capacity, leaving little or none remaining for processing any additional visual information [15]. Consequently, high perceptual load contexts tend to prevent the processing of unexpected and task-irrelevant information (e.g. alarms) and facilitate various forms of inattentional blindness [16]. However, this propensity to remain unaware of unexpected, though fully perceptible stimuli, is not limited to vision. Indeed, unexpected salient sounds can remain unnoticed under attention-demanding settings [17–20]. Although less well-known than its visual counterpart, this inattentional deafness phenomenon [21] is likely to have important consequences for safety-critical situations as pilots often have to deal with auditory alarms whilst being overloaded with various information. In the literature, two experimental paradigms address this failure of attention: the inattentional deafness paradigm and the interference paradigm. In the inattentional deafness paradigm, the ability to perceive an unexpected sound during a task is directly assessed by the participant’s behavioral response to it. In the interference paradigm, the subject is asked to ignore a distractor stimulus while performing an ongoing task. The available resources of the attentional system are measured by the level of interference caused by the distractor stimulus on the ongoing task.

Interestingly, Macdonald and Lavie [22] demonstrated in the inattentional deafness paradigm that participants involved in a visual discrimination task were subject to inattentional deafness. The inattentional deafness rate was further increased when the visual task involved a high level of perceptual load compared to a moderate perceptual load. It appears that engaging in a task under high perceptual load may lead to a decline in the probability of processing an auditory stimulus. While this previous study concentrated on the influence of perceptual load on inattentional deafness, some other researchers focused on the effect of cognitive load by using the interference paradigm. For example, Berti and Schröger [23] analyzed how cognitive load in working memory alters the vital automatic attentional orientation toward unexpected stimuli by using an interference paradigm. The behavioral results showed that the ability to automatically process supplementary auditory stimuli (distraction effects) was still present but reduced markedly with higher task demands. SanMiguel, Corral, & Escera [24] found similar results: distraction caused by novel sounds was reduced in a 1-back working memory condition versus a no-memory control condition. According to the authors, involuntary attention allocation is under the control of top-down mechanisms that are affected by cognitive load.

Classically, the inattentional deafness paradigm manipulates perceptual load, whereas the interference paradigm often manipulates cognitive load. For example, in aeronautics, the complexity of cockpits generates a high cognitive load and alarms require an active immediate response. Therefore, we combined an inattentional deafness paradigm, where explicit responses to alarms have to be made, with cognitive load variations (manipulation generally used in interference paradigm).

### Uncovering the neural correlates of inattentional deafness

The brain activity involved in processing auditory information has been extensively studied using Electroencephalography (EEG) techniques to measure Event-Related Potentials (ERPs), even in an aeronautical context [25–28]. The P300 component, one of the most commonly studied ERPs, reflects the detection of an expected but unpredictable target (the oddball) in a stream of stimuli [29]. It can be elicited by the “oddball” paradigm. The P300 is typically observed in a time window between 300 to 600 ms after the auditory stimulus onset and reflects the occurrence of cognitive and attentional processes (c.f. [29] and [30] for a detailed review). When attentional focus deviates from the target, the P300 amplitude significantly decreases [31]. This link indicates that the P300 component is an excellent candidate to determine whether an auditory stimulus has broken through the attentional barrier. Importantly, it is generally accepted that a distinction can be made between two subcomponents of the P300, namely the novelty P3 and the target P3 (also called P3b, or “classical” P3). Novelty P3 is a large positive deflection with a fronto-central scalp distribution that is elicited by novel, non-target stimuli and that mainly reflects involuntary attention shifts to changes in the environment [32,33]. It is functionally related to another subcomponent called P3a, that seems to be more specifically related to deviant auditory non-target events [34]. In contrast, the P3b, has a more posterior-parietal scalp distribution and a somewhat longer latency than novelty P3 and P3a. The P3b has been regarded as a sign of processes of memory access that are evoked by evaluation of stimuli in tasks that require some form of action like a covert or overt response, ecologically closer to a real alarm occurring in a cockpit [35].

Several studies have revealed that the auditory P300 amplitude might be lower in audio-visual dual-tasks compared to a singular auditory task [36–39]. Auditory ERPs modulations by cognitive load were also observed [23,40,41]. In those studies, the researchers recorded auditory ERPs responses to deviant tones while participants had to perform a visual task (interference paradigm).The reduced openness to distractors with increasing cognitive task load reduced the P3a amplitude, i.e. less attentional resources were available to perceive unexpected stimuli, potentially leading to inattentional deafness.

Another commonly studied ERP is the auditory N100. This component is considered to reflect auditory cortices activity (eg. [42]) and early analysis of stimuli characteristics such as intensity [43]. Traditionally, considered too early to be impacted by top-down influences, several studies showed that some cognitive processes, such as attention [44], can also affect its amplitude. Qu et al. [45] showed that auditory N100 evoked potential following task irrelevant auditory clicks was affected by the visual working memory load during a face recognition task. According to the authors, auditory and visual pieces of information interact in the prefrontal cortex, which in turn modulates working memory processes and selective attention in a cross-modal manner.

To the best of our knowledge, the relationship between the inattentional deafness phenomenon and P300 amplitude has never been explicitly investigated. The analysis of both N100 and P300 component modulations can indicate whether variations of cognitive load in a given task affect early pre-attentional or later attentional processes.

## Objectives

There are three main objectives in this work. Firstly, we aimed at designing an experimental paradigm that allowed a sufficient number of occurrences of the inattentional deafness phenomenon within each participant as to reach a satisfactory statistical power to assess its effects at the electrophysiological level. In the Macdonald and Lavie study [22], only one occurrence of the inattentional deafness phenomenon was successfully reproduced, further attempts failing due to the impact of the pre-exposure to the sound. Secondly, we attempted to observe the occurrence of inattentional deafness with respect to contextual variation in cognitive load. Interleaved low load and high load trials would be closer to an ecological piloting situation and would prevent differences in the overall level of motivation, attentional set, vigilance, task engagement, or performance strategies, which are likely to occur when participants perform long series with the same load context (i.e. block design). Finally, we strived to examine the possible concomitance between inattentional deafness and P300 amplitude modulations. For this purpose, we performed EEG measurements during a simplified but plausible aviation decision-making task in which participants were asked to take into account both visual and auditory signals. Auditory signals were based on the oddball paradigm.

To summarize, the experiment’s key points are the following: we aim to test 1) whether an increase in cognitive load provokes a decrease in the alarm detection rate (deviant sounds), and 2) which of the ERP components (and corresponding processing stages) are affected by cognitive load.

## Method

### Ethics Statement

The experiment received the ethics committee approbation CERUL (Comités d'Ethique de la Recherche avec des Etres Humains de l'Université Laval) 2010–028. Participants were given full information about the experimental protocol, received monetary compensation, and an informed written consent was obtained before participation.

### Participants

Sixteen right-handed healthy male volunteers (mean age = 20.9 years, SD = 1.22) were recruited at Institut Supérieur de l’Aéronautique et de l’Espace (ISAE) for this study. All participants were French undergraduate students; none of them had a history of neurological disease, psychiatric disturbance, substance abuse, or taking psychoactive medications. One participant was excluded because he demonstrated an alarm detection rate below 95% during the oddball control condition. All participants went through the same procedure, which began with a fatigue assessment using Pichot’s fatigue scale [46]. Participants with a score over 22 (excessively tired) would have been excluded. However, all scored below this threshold. Next, they performed the Paced Auditory Serial Addition Test (PASAT, [47]) to assess their working memory performance. No participant obtained a prohibitive score in this test (below 35% for the PASAT 3” or below 23 for the PASAT 2”). Finally, they completed a laterality test (Edinburgh Handedness Inventory, [48]) to confirm that they were right handed. This was a necessity with the experimental set up.

### Tasks

Two different tasks were used in this study: an aviation decision-making task (landing task) and an auditory task (oddball task). The combination of both tasks is called *dual-task scenario*. The unique performance of the oddball task is named *control scenario*. The landing task had two levels of difficulty: interleaved low load trials and high load trials. We thus defined three load conditions: the control condition, the low load condition and the high load condition.

**Fig 1:**
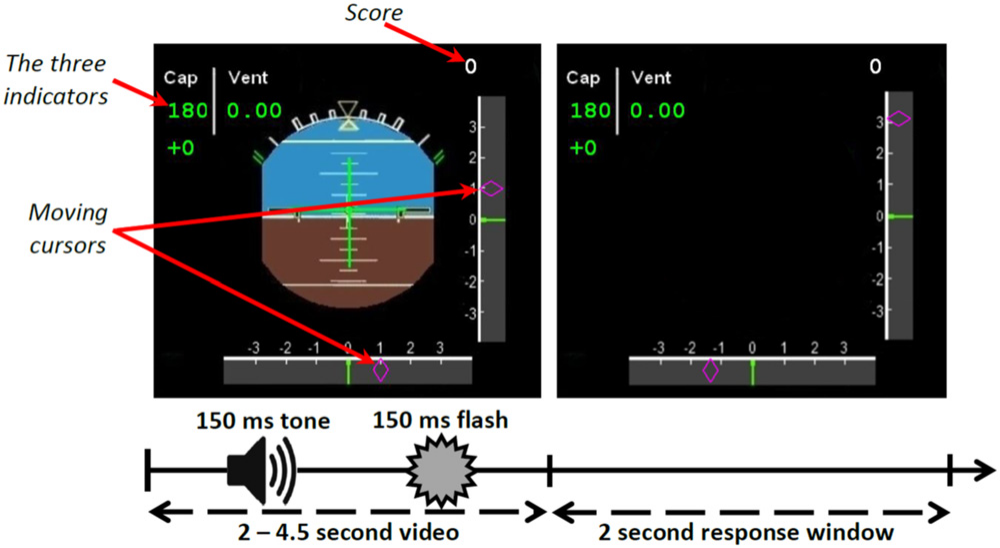
Diagram of the scenarios’ process. First a 2 to 4.5-second video was displayed, during which a sound was played and a colored circle displayed, followed by a 2-second response time window. The 3 indicators are in the upper left corner, the score to the landing task in the upper right corner.

**Fig 2:**
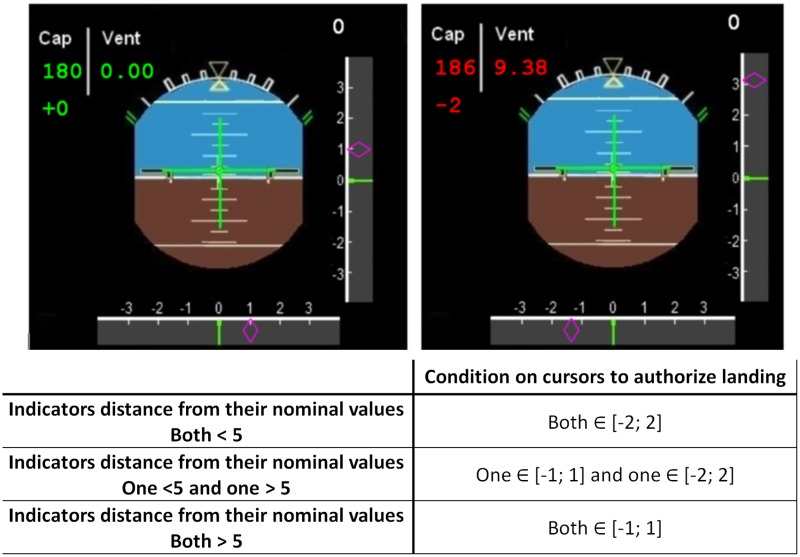
Illustration of the two landing task difficulty levels and the table of rules for landing. On top, a low load landing task video on the left, and a high load landing task video, on the right. In the high load condition, indicators appeared in red. Below, the rules for deciding whether it was possible or not to land in the landing task.

**The landing task.** Correct response rates in landing and visual alarm detection rates were the dependent variables. The participants were submitted to a series of trials that consisted of a 2 to 4.5 second video clip reproducing a landing situation, followed by a 2 second response time window during which they had to respond if they would authorize the landing or not by pressing a button (see Fig. 1). During the videos, a Primary Flight Display (PFD) inspired from plane cockpits was presented with various indicators: the heading (“Cap”), the magnetic declination, and the wind speed (“Vent”) were located in the upper left corner. The magnetic declination had to be added to the heading. All these indicators were static during the video. In addition, two moving cursors representing an Instrument Landing System (ILS) were displayed: one on a vertical axis and the other on the horizontal axis. During the response window, the PFD instrument was removed to indicate the beginning of the response window, and the ILS cursors were frozen but still displayed on the screen. In order to provide the participants with complete information for the landing decision, all other indicators were also displayed. Importantly, participants were asked to decide whether the landing was possible or not according to the indicators and the final position of the cursors. Fig. 2 presents the rules for landing decision depending on the cursors and indicators. The rules varied depending on the cognitive load level of the trial. In the low-load trials, the indicators located on the upper left corner appeared in green and did not show any potential problems. They clearly indicated a perfect nominal heading, no magnetic deviation, and no wind. Hence, the decision to land relied solely on the two cursors, which had to be located between-2 and +2 on the arbitrary scale. In the high-load trials, the indicators’ values appeared in red, i.e. they showed a degradation of the aircraft status. The distance between the two cursors and the center of their respective axes became more conservative the larger the deviation of the indicators from their nominal values (Fig. 2). During the response time window, participants responded by pressing the response button for possible landing (right) or no landing (left).

To further increase the visual load associated with the landing task, a colored circle was displayed for 150 ms during both low load and high load trials, at a random time, during the first time window. This circle was either green (probability = 0.9) or red (probability = 0.1) and equally likely to be displayed on the right or on the left of the screen during all cognitive load trials. A red circle represented a visual alarm. The participants were asked to report this alarm during the response window, after answering to the landing task, by pressing the central button of the response box.

An ongoing tally of the correct landing task score was displayed on the upper right corner of the PFD (see Fig. 1). For each correct landing response, the score increased by 1 point. When participants missed a response, or made an incorrect response, this score remained the same (no addition or subtraction). The score system was used to engage and motivate the participants. Low and high load trials of the landing task were interleaved throughout the trial series and were equally likely to occur. We did not change the design of the auditory alerts across the levels of difficulty of the landing task. Changing the design of a sound may result in the attentional set being more or less clearly focused on some characteristics of the sound [49]. Finally, we chose to interleave the two cognitive loads conditions within the same run instead of creating blocks of the same difficulty. This decision prevented the use of response strategies as the participants could not predict the load of the next trial. The random variation of the cognitive load within the same run is close to an ecological piloting situation where the occurrence of alerts is unpredictable.

**The oddball task.** This task consisted of a classical oddball sound series. Reaction times (RTs) and percentage of correctly reported deviant tones were the dependent variables in this task.

During the video clip of the landing situation, one of two 50 dB (SPL) tone types lasting 100 ms was randomly played between 500 ms after the start and 500 ms before the end of the first time window. The tone was either standard (frequency = 1900 Hz, *p* = 0.8) or deviant (frequency = 1950 Hz, *p* = 0.2). The frequencies were chosen from the study of P300 components conducted by Kolev et al. [50]. The tones were clearly distinctive and perceptible from the white noise (as assessed during the control condition). Participants were asked to report the alarm by pressing a button during the response-window following the short video. They were asked not to press any buttons if the standard tone was played. During the response window, participants were instructed to respond to the landing task at first, and then to report the potential alarm. The 2-second response window elapsed independently of the response. We separated the first time window from the response time window to avoid all motor responses when tones were played. A white noise at 50 dB (SPL) was played continuously during the task. The deviant tones were never played when a red circle (visual alarm of the landing task) was displayed in the same trial. As a consequence, only one critical event (a deviant tone or a red circle) occurred and had to be reported per trial.

### Scenarios

Participants had to go through two scenarios: the first one was the dual-task scenario, during which they had to perform both tasks (landing task and oddball task simultaneously), and the second one was the control scenario, during which they only had to perform the oddball task while the landing task was displayed but to be ignored.

**The dual-task scenario.** Participants responded to a set of 400 trials. This scenario assessed the participants’ ability to detect the deviant tones (oddball task) while performing the landing task in parallel. To avoid effects of attentional set, an equal emphasis was placed on the aviation decision-making task and the classical oddball task across the two levels of difficulty. Attentional set is a preparatory state of the information processing system that prioritizes stimuli for selection based on attended features [51]. It is linked to endogenous (top-down) processes [52]. Manipulation of the task load is supposed to exert exogenous (bottom-up) effects on participants’ performances. Attentional set may act as a confounding factor if different instructions are provided with respect to the processing load level—participants may be inclined to focus more on one of the concurrent tasks at the expense of the other task. In this case, the diminution of performance on the other task may be related to a reduced priority rather than differences in the availability of processing capacity.

**The control scenario.** In the control scenario, participants responded to a set of 200 trials to obtain their nominal behavioral and electrophysiological responses to the auditory stimuli. The landing task was also displayed on the screen but participants were instructed not to pay attention to the landing task, but rather to fixate on the central green cross of the PFD and to report the deviant tones (oddball task).

### Procedure

**Fig 3:**
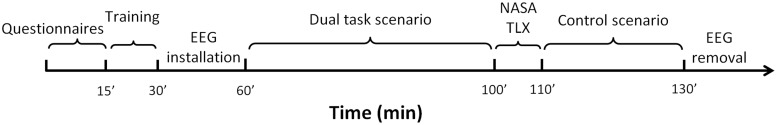
The procedure timeline.

The whole procedure lasted 150 min, including 30 min to install the EEG sensors and 60 min of EEG recording (Fig. 3). First, we asked to complete the behavioral questionnaires (15 min). Next, participants were trained for the three experimental conditions using two sets of 20 trials. Here, we trained participants for all possible situations that could occur during the experiment. Importantly, participants had to demonstrate their ability to detect each type of alarm (auditory and visual). Once competency was achieved, the EEG electrode cap was placed as well as the Electro-OculoGraphic (EOG) electrodes (blinks or saccades detection). In the main experiment, participants were seated in a comfortable reclining armchair, placed in a dimly lit, in a sound-dampened room. They were instructed to keep their forearms stable on the chair’s arms, with their two hands resting on the central response pad. The dual-task scenario had 400 experimental trials and lasted 40 minutes. Immediately after the main experiment, participants filled out the NASA Task Load Index (NASA TLX, [53]) questionnaire. This questionnaire gave us an evaluation of the subjective mental demand elicited by the dual task. Finally, participants finished the experiment with the 200 trials control scenario (20 min).

### EEG recordings and pre-processing

EEG data was continuously recorded with a Biopac EEG system (*BIOPAC* Systems, Inc. 42 Aero Camino Goleta, CA 93117). Prior to the experiment, a 19-electrode cap (CAP100C) was placed on the participant’s head. Eight electrodes sites were recorded, distributed throughout the brain volume according to the International 10/20 system: Fz (frontal), Cz (central), Pz (parietal), T3 and T4 (temporal), O1 and O2 (occipital), and the ground electrode. The Biopac was connected by parallel cables with the experimental computer in order to mark trial onsets on EEG data. Three EOG electrodes were placed to the left, right, and above the eyes. Artifacts created by eye movements were removed in real-time from the cerebral signal with *Acqknowledge* 4.0 (*BIOPAC* Systems) EOG artifacts removal function. Impedance of the cap electrodes was kept below 5 kΩ. Signals were recorded with a 0.1 Hz high-pass filter (6 dB/Oct) and a 49 Hz low-pass filter (18 dB/Oct). The sampling rate was 500 Hz with a 16-bit A/D conversion.

### Offline data processing

EEG data analysis was performed using EEGLAB 11.0.3.1b [54] running under MATLAB 7.1 (The Mathworks, Inc.). EEG signals were segmented in each trial to obtain epochs starting from 200ms before the stimulus onset until 1000ms after stimulus (baseline-200 to 0 ms). The EEG was low-pass filtered at 30 Hz (IIR, attenuation: 40 dB, transition interval: 1 Hz) filter. Data rejection was performed to remove epochs not fitting in a [-100 µV, 100 µV] window. For the low load dual-task condition, 200 ERP trials were administered for each participant, and a mean of 182.2 trials were retained after artifact rejection. For the high load dual-task condition, 200 trials were administered for 183.4 retained, and for the control condition, 200 trials were administered for 183.8 retained. Averaged ERPs were then independently calculated for each channel and condition. The time window for the average amplitude of the P300 was determined both from visual inspection and from results of significant one-way ANOVA (factor “type of tone”, standard vs. deviant tones) of consecutive 50-ms latency windows from 370 to 620 ms post stimulus, resulting in a 520 to 570 ms time window. The same methodology was applied to the initial time window, 100 ms to 250 ms post-stimulus for the N100 component, with no 50-ms window showing a significant result. For subsequent N100 analyses the final time window for the N100 component was 100 ms to 250 ms (according to literature and visual inspection).

### Statistical analysis

All data was analyzed with Statistica 7.1 (StatSoft). Mean percentage of correct responses to the landing task, mean percentage of unreported auditory alarms, the false alarm rate as well as mean reaction times (RT) for the auditory alarm detection were analyzed. In addition, mean auditory N100 and P300 amplitudes were calculated for the three task conditions. No ERPs analysis was performed on the visual stimuli. Finally, NASA TLX scores on a range of 1–20 were collected after the participants had completed the dual task.

**Behavioral analyses.** We performed Kolmogorov–Smirnov goodness-of-fit test in order to test against normal distribution for the percentage of alarm detection within the control and the dual-task scenarios. Since both variables were not normally distributed, we chose non-parametric tests (Friedman’s ANOVA and Wilcoxon signed-rank tests for post-hoc comparison) to examine the effects of the cognitive load on the alarm detection rate.

Correct response rate of the landing task was assessed by a 2 x 2 ANOVA with within-subjects factors “cognitive load” (low vs. high) and “type of tone” (standard vs. deviant). The main goal of this analysis was to ensure that there was a significant difference between both cognitive load conditions (low vs. high), which would confirm successful manipulation of the difficulty. If those assumptions were met, then the performance would be higher in the low load condition than in the high load condition. To analyze the potential effect of time on the participants’ performance over the duration of the experiment within the dual-task scenario, a complementary analysis was conducted. For this analysis, the mean rates of unreported tones for the 40 low load trials and the 40 high load trials were divided into two groups: the first and last twenty trials of the dual task scenario. We then performed a Wilcoxon signed-rank test comparing the alarm detection performance in the first half with the performance in the second half of the scenario.

**NASA TLX.** We administered the NASA TLX immediately at the end of the dual-task scenario to examine which of the six mental workload subscales (mental demand, physical demand, temporal demand, performance, effort, and frustration level) was the most exerted by our task.

**ERP analyses.** While seven electrodes sites were recorded, ERPs analyses were focused on the three midline electrodes (Fz, Cz, Pz). A two-step analysis was conducted for each ERP component of interest. First, we focused only on the control scenario. For this data, a three-way repeated measures ANOVA (2 x 2 x 3) with within-subjects factors “load” (low vs. high cognitive load of the landing task), “type of tone” (standard vs. deviant) and “electrode” (Fz vs. Cz vs. Pz) was used. Its purpose was to determine the best electrode site for the ERP component, and the absence of effect of the load in the control scenario, ensuring that participants were not doing the landing task in both low load and high load landing task trials. Regarding the dual-task scenario, we focused on the effect of cognitive load on the alarm-related ERP amplitudes. For this purpose, a two-way ANOVA was conducted with the three experimental conditions (control condition vs. low cognitive load dual-task condition vs. high cognitive load dual-task condition) on the three midline electrodes. Tukey’s HSD tests were used as post-hoc tests.

## Results

### Behavioral results

**NASA TLX.** The mental demand dimension was most exerted by the dual task, with a mean rating of 14.27 (SD = 2.69), 1 representing a low mental demand and 20 a high mental demand. The other dimensions were rated as follow: physical demand: M = 8.00, SD = 6.32; temporal demand: M = 12.07, SD = 4.15; performance: M = 7.73, SD = 2.58; effort: M = 13.2, SD = 3.80; frustration: M = 9.20, SD = 5.10. We performed a one-way ANOVA with within-factor “dimension”, there was a significant main effect (*F*(5, 70) = 7.19, *p* <. 001, *η^2^p* = .34). Tukey’s HSD test revealed that mental demand was rated significantly higher than frustration (HSD, *p* <. 05), physical demand (HSD, *p* <. 01) and performance (HSD, *p* <. 001). The effort dimension was significantly higher than physical demand (HSD, *p* <. 01) and performance (HSD, *p* <. 01). Temporal demand was significantly higher than performance (HSD, *p* <. 05).

**Landing task performance.** The correct response rate to the landing task in the low load condition (M = 96.4%, SD = 2.7%) was higher than the correct response rate in the high load condition (M = 90.0%, SD = 3.6%), *F*(1, 14) = 91.60, *p* <. 001, *η^2^p* = .87. This suggests that the difficulty was successfully manipulated. There was no difference in performance between trials with the standard tone (M = 93.2%, SD = 3.5%) and trials with the deviant tone (M = 93.8%, SD = 2.1%), *p* = .54, and no significant interaction between these two variables, *p* = .97. Finally, we observed a decrease in the detection rate of the visual target with increasing load. This effect corresponded to a significantly higher detection rate in low load trials (M = 87.7%, SD = 13.6%) than in high load trials (M = 75.0%, SD = 21.0%), *F*(1, 14) = 7.10, *p* <. 05, *η^2^p* = .34.

**Fig 4:**
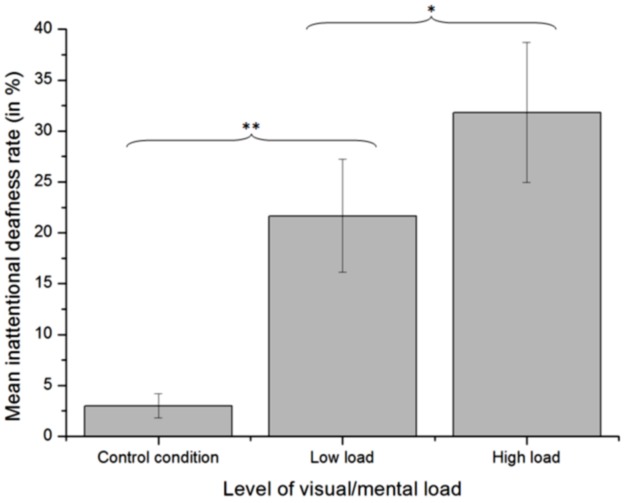
Mean inattentional deafness rate across the three levels of cognitive load. Error bars represent the standard error of the mean. *significant difference at *p* <. 05. **significant difference at *p* <. 001.

**Auditory alarm detection**. First, the mean rate of unreported deviant tones increased with the load of the landing task (Friedman’s ANOVA: χ²(15) = 22.107, *p* <. 001). It was lower in the control condition (M = 3.0%, SD = 4.7%) than in the low load condition (M = 21.7%, SD = 21.5%), Z = 3.180, *p* <. 001, and it was lower in the low load condition than in the high load condition (M = 31.9%, SD = 26.6%), Z = 2.197, *p* <. 05, as shown in Fig. 4. The signed-rank test also indicated that the inattentional deafness rate in the control condition was significantly lower than in the high load condition (Z = 3.474, *p* <. 001).

Second, the RTs to report the rare auditory target were significantly lower in the control condition (M = 0.530 s, SD = 0.124 s) than in low load (M = 1.047 s, SD = 0.215 s) and high load trials (M = 1.170 s, SD = .0362 s), *p* <. 001 for both comparisons. Finally, there was no significant difference in the reaction times between low load and high load trials (*p* = .38).

Third, the signed-rank tests showed no difference in the mean rate of unreported deviant tones across time, either for the low load (20 first trials: M = 21.7%, SD = 21.0%; 20 last: M = 20.0%, SD = 24.0%, *p* = .50) or the high load condition (20 first trials: M = 35.0%, SD = 30.9%; 20 last: M = 28.7%, SD = 26.4%, *p* = .33).

Finally, the false alarm rate, measured for both visual and auditory targets, showed no significant difference between low load (M = 3.2%, SD = 2.1%) and high load trials (M = 3.8%, SD = 2.1%) in the dual task (signed-rank test: *p* = .22).

### N100 results

**Control scenario.** The ANOVA revealed no significant effect of the type of tone on the N100 amplitude (*F*(1, 14) = 0.15, *p* = .70), and a significant effect of the electrode site (*F*(1, 14) = 18.35, *p* <. 001, *η^2^p* = .58). Tukey’s HSD test revealed that N100 amplitude at Fz (M = -3.28 µV, SD = 3.56 µV) was significantly bigger than at Cz sites (M = -2.72 µV, SD = 2.69 µV) (HSD, *p* <. 05), and Pz (M = -2.02 µV, SD = 2.61 µV) (HSD, *p* <. 001). Given those results, we chose the Fz site for the subsequent N100 analysis. In addition, there was no significant effect of the load of the landing task on the N100 amplitude (*F*(1, 14) = 0.02, *p* = .90), justifying the merging of the two load conditions into one “control condition” for the following N100 analysis.

**Effects of the cognitive load on the N100 amplitude.** This analysis still revealed a significant main effect of the electrode site (*F*(2, 28) = 6.57, *p* <. 01, *η^2^p* = .32), no main effect of the load (*F*(2, 28) = 0.65, *p* = .53) and no electrode * load interaction (*F*(4, 56) = 0.70, *p* = .59).

### P300 results

**Control scenario.** In line with the behavioral analysis, the 2 x 2 x 3 ANOVA with within-subjects factors “load”, “type of tone” and “electrode” revealed that the load factor had no significant effect over the P300 amplitude (*F*(1, 14) = 0.02, *p* = .89), which confirms that participants did not process differently low and high load trials in the control scenario, and support our decision to merged those two load conditions into one “control condition”. A significant main effect was found for the electrode site (*F*(1, 14) = 21.19, *p* <. 001, *η^2^p* = .62): the P300 peak was higher at Pz (M = 2.75 μV, SD = 6.36 μV) than at Fz (M = -1.641 μV, SD = 7.96 μV) (HSD, *p* <. 001), and at Cz recording sites (M = 0.65 μV, SD = 6.38 μV), (HSD, *p* <. 05). In addition, there was a significant electrode * type of tone interaction (*F*(2, 28) = 11.66, *p* <. 001, *η^2^p* = .45). As expected, Tukey’s HSD test revealed that the effect of the type of tone was higher at Pz with rare tones eliciting a higher P300 (M = 6.08 μV, SD = 7.32 μV) than standard tones (M = -0.57 μV, SD = 2.42 μV) (HSD, *p* <. 001). Based upon the previous results, we focused subsequent analysis of load manipulation on rare tones.

**Fig 5:**
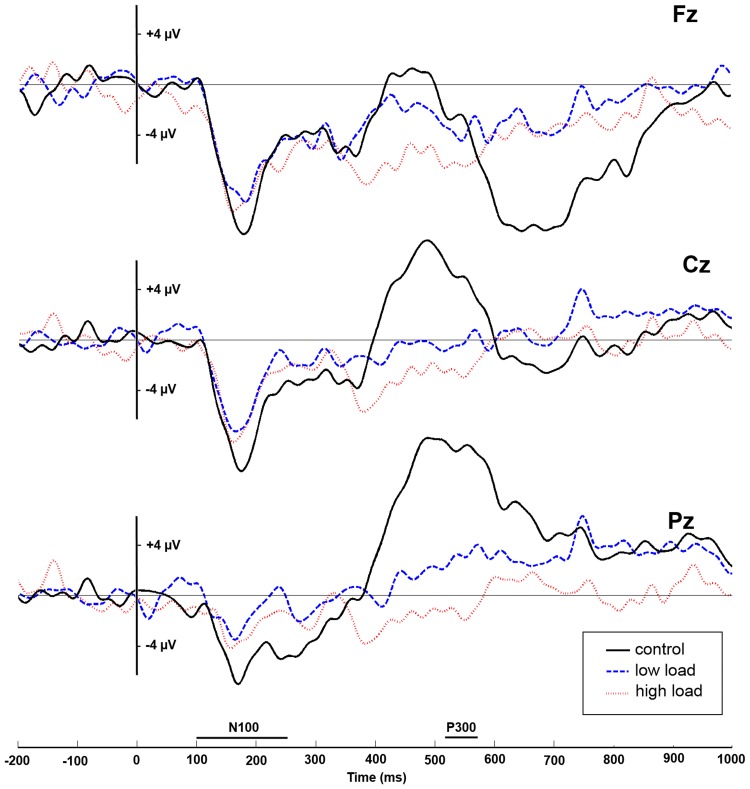
Mean ERPs and mean P300 amplitudes for the three levels of load on the three midline electrodes. For each electrode, the horizontal axis denotes time in ms, with the N100 and P300 time-windows used for analysis indicated, and the vertical axis denotes amplitude in µVolts.

**Effects of the cognitive load on the P300 amplitude.** This analysis still revealed a significant main effect of the electrode site (*F*(2, 28) = 15.69, *p* <. 001, *η^2^p* = .53), no main effect of the load (*F*(2, 28) = 2.14, *p* = .14) and a significant electrode * load interaction (*F*(4, 56) = 9.72, *p* <. 001, *η^2^p* = .41). Tukey’s HSD test post-hoc analyses showed that at the Pz site, the P300 observed in the control condition (M = 6.08 μV, SD = 7.32 μV) was higher than in the low load condition (M = 1.62 μV, SD = 4.58 μV) (HSD, *p* <. 001); and the P300 in the low load condition was higher than in the high load condition (M = -0.76 μV, SD = 2.62 μV), (HSD, *p* <. 05), as shown in Fig. 5.

**Fig 6:**
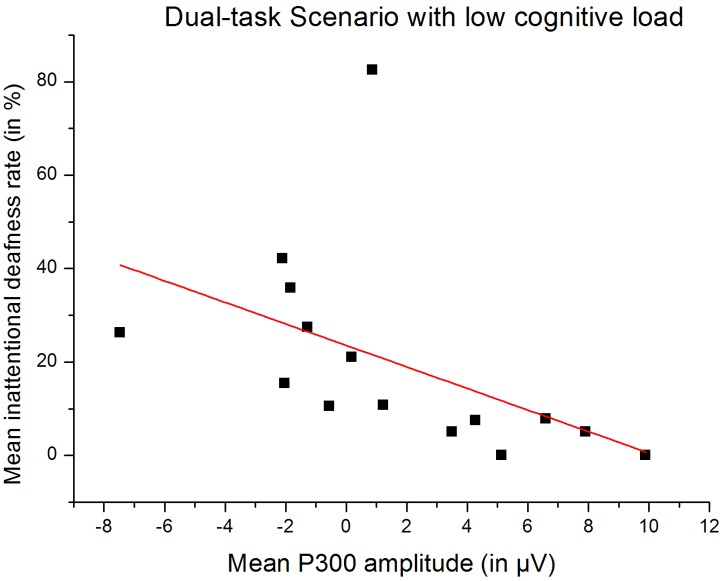
Correlation between the mean inattentional deafness rate and the mean auditory P300 amplitude. On the horizontal axis, the mean P300 amplitude during the dual task scenario, in µV. On the vertical axis, the mean inattentional deafness rate, during the dual task scenario (*p* <. 01). Linear regression intercept: 23.57, slope: -2.30.

**Correlation between unreported deviant tones rate and P300 amplitude.** We examined the Spearman rank order correlations between the individual P300 amplitude elicited by the deviant tones and the individual rate of unreported deviant tones, in the low and high load conditions. The correlation was significant in the low load condition (r = -.79, *p* <. 001) meaning that participants with a high unreported deviant tones rate also had low P300 amplitudes (Fig. 6). The correlation was not significant in the high load condition (r = -.45, *p* = .09). We calculated the same correlations removing the outlier (low load condition, rate of unreported deviant tones: 82.5%, P300 amplitude: -4.11 µV). The results remained the same: a significant correlation in the low load condition (r = -.87, *p* <. 001) and no correlation in the high load condition (r = -.33, *p* = .25).

## Discussion

Our study aimed at studying the links between ERPs amplitudes and the inattentional deafness phenomenon by creating an experimental paradigm in which the inattentional deafness phenomenon could occur numerous times for each participant. We designed a simplified landing task with two levels of cognitive load (high vs. low). To study the effect of contextual variation in the cognitive load and prevent changes in motivation, vigilance, priorities or strategy, low load and high load trials were interleaved. While participants were subject to this task, an oddball paradigm was simultaneously administered to assess their ability to detect an alarm (i.e. the rare deviant sound in the oddball paradigm). We hypothesized that variations in cognitive load would alter the participants’ ability to detect alarms. In addition, we assumed that the occurrence of inattentional deafness would be concomitant with an alteration of ERP components, and that this alteration would be proportional to the cognitive load level. An effect of the cognitive load on the auditory N100 amplitude would indicate an alteration of pre-attentive or early attentive components whereas alteration of the P300 (more specifically the P3b) would indicate an alteration of mechanisms related to voluntary orientation of attention.

The behavioral results showed that our task was successful in provoking inattentional deafness. Indeed, the NASA TLX results suggested that participants used a substantial amount of cognitive resources (mental demand dimension) in the task, favoring the occurrence of inattentional deafness and thereby validating the experimental procedure. As low and high cognitive load conditions were interspersed through the runs, their respective mental demands could not be compared. In the control scenario (oddball task alone), only 3% of the alarms were not reported by the participants. In the low load condition of the dual-task scenario, 21.7% of the alarms were missed when the low load dual-task was processed. This number significantly increased to 31.8% in the high load condition of the dual-task scenario. This result showed that cognitive load can provoke inattentional deafness. This result is consistent with previous work of Macdonald and Lavie [22] on the impact of perceptual load. Importantly, the comparison between the control and the dual-task scenarios should be considered with caution. In addition to a higher cognitive load in the dual-task scenario than in the control scenario (auditory oddball), a response competition occurred between the landing task and the auditory task. This competition did not exist in the control scenario. Therefore, the comparison between the low and high load condition in the dual-task scenario must be considered as the most important analysis. Furthermore, unreported alarms can reasonably be considered as the result of inattentional deafness rather than the consequence of another phenomenon altering the response rate of the participants, such as changes in the participants’ priority modality (landing task or oddball task) or vigilance depending on the level of cognitive load. Indeed, the false alarm rate was very low and remained constant in both cognitive load conditions, contrary to the decreased rate of reported alarms with respect to the increased load. Those results indicate that participants did not simply change their response criterion level or were more focused on the landing task, which would have led to a decrease in both the reported alarm rate and the false alarm rate. The increasing number of unreported alarms is arguably due to an inattentional deafness increasing with cognitive load. The response times were also the same in both cognitive load conditions, i.e. the reduced reported sound rate in the high load condition was not related to the limited response time.

The effect of the cognitive load on the ability to detect an alarm was also significant on electrophysiological measures, as showed by the variation in amplitude of the P300 ERP component. ERP analysis showed that a drastic diminution of the auditory P300 amplitude was concomitant with the increased occurrence of inattentional deafness. Auditory P300 amplitude diminished in the landing task with low load condition in comparison to the control condition and further diminished with an increase of the load (high load vs. low load). These results tend to confirm that our aviation landing task left few resources to process auditory alarm in the low-load condition and even fewer in the high-load condition. FMRI studies of oddball paradigms have identified a “target detection network” involving inferior parietal [55–57] and frontal generators of the P3b [58–60]. According to Bledowski [57], the involvement of these cortical areas in the generation of the P3b can be integrated in Kok’s model of the P3b potential [61]. In this model, the P3b amplitude reflects the cognitive capacity invested in the categorization of task-relevant or significant events, controlled by the joint operation of attention and working memory. Thus, even if participants were asked to allocate similar priority to both tasks, the visual dominance and the complexity of the landing task may have led to an implicit prioritizing rule, leaving less available resources for the auditory target detection within the aforementioned cortical structures. This P300 diminution could be used as a precursor to determine the susceptibility of an individual to alarm misdetection.

Interestingly, this susceptibility to alarm misdetection along with P300 modulation might be in line with other studies using the interference paradigm [23,40,41]. Those studies interpreted reduced P300, provoked by an increased cognitive load, as a decreased “openness” of the attentional system to distractors. Our results and these studies suggest that a high cognitive load leads to an extremely reduced openness of the attentional system which encourages the participants to neglect the deviant tones. Yet, it is vital to be able to take into account unpredictable environmental changes, even when the cognitive system focuses on another task. For example, we have to be able to hear an approaching car when crossing the street while using a phone at the same time.

In our study, the inattentional deafness rate did not significantly diminish with time-on-task. This result suggests a robust and sustained effect of load on the ability to perceive unpredictable auditory stimuli. In addition, further analysis showed that individual mean P300 amplitude was correlated with the individual inattentional deafness rate. It gives additional evidence on the link between the inattentional deafness occurrence and the P300 potential. Importantly, we assumed that the considered subcomponent is the P3b (rare event related P3). This is supported by the fact that participants have to respond to the deviant tones in all cases. Indeed, the P3a seems to be more specifically related to deviant auditory non-target events [29,34,57] with a more fronto-central distribution. In contrast, the P3b has a more posterior-parietal scalp distribution and a slightly longer latency than P3a. P3b has been regarded as a sign of processes of memory access that are evoked by evaluation of stimuli in tasks that require a covert or overt response (eg. [35]).

Finally, the absence of effect on the N100 amplitude suggests that the cognitive load did not alter early sensory processes and had only an impact on late attentional process. One could expect that visual dominance would have involve sensorial gating mechanisms [27,45] in such task; however, in the present study we manipulated the load in terms of cognitive demand and not perceptual complexity which may have led to attentional but not pre-attentional effects. In addition, the fact that cognitive load altered the P300 rather than the N100 suggests that cognitive load had an impact on the voluntary orientation of attention rather than a more general and non-task specific impact. Indeed, P3b is known to index the maintenance and control of ongoing information [62] requiring some form of overt response.

Taken all together, behavioral and EEG results support the hypothesis that the inability of pilots to react to some auditory warnings in the cockpit during critical flight phases is not voluntary but rather associated with cognitive and attentional limitations.

There are several limitations in the present study. One limitation is the difference between the detection of a 50 Hz change between single sounds (frequencies chosen from [50]) and the salient acoustic alarms in a real cockpit. In order to observe and reproduce numerous occurrences of inattentional deafness, we were compelled to increase the discrimination difficulty between the deviant and the standard tones. However, the deviant tones were clearly distinctive from the standard tones and from the white noise, as showed by the high level of detection during the control condition (i.e. 97% of detection). In addition, the ambient noise level in the cockpit is considerably high (engine, wind noise, communications etc). Experiments in flight simulator showed that even salient realistic alarms can be unnoticed in high cognitive load context [63]. In consequence, increasing the signal-to-noise ratio is certainly not sufficient to efficiently prevent the inattentional deafness phenomenon in aviation.

Another limitation of the study is the lack of significant correlation between the inattentional deafness rate and the P300 amplitudes in the high cognitive load condition (while this correlation was significant in the low cognitive load condition). We arguably state that this may be explained by the suppression of the P300 component and thus its lack of variation among the participants in the high vs. low load condition.

One also could claim that the inability to detect the alarm was related to the expiration of elements within short-term memory rather than perceptual and cognitive limitations. For example, Friedman & Trott [64] showed that variations in P300 amplitudes may be an indicator of whether an item will be recollected or not. However, in Friedman‘s experiment, the restitution phase took place separately and significantly after the phase of encoding an item. To circumvent this short-term memory problem, participants within this experiment were asked to immediately report each alarm they perceived during the response window (mean delay between the alarm and the response window onset = 3.25 s). Under such conditions, we limited the possibility that participants would simply forget they heard the alarm before needing to report it.

Finally, future studies could focus on modulating separately perceptual and cognitive loads, to identify their respective roles on the inability to detect auditory warnings. Source localization could be also considered for further analyses of the cognitive process involved in inattentional deafness. This technique would help to clarify the relationship between ERPs characteristics and the attentional state of a pilot in situations that are likely to provoke inattentional deafness.

## Conclusion

The major contribution of this study lies in the co-occurrence of inattentional deafness during an aviation task and alterations of the P300 (P3b) ERP, a component associated with the voluntary orientation of attention. While inattentional deafness has been studied through oral responses following a single trial [21,22], we developed a paradigm inducing multiple occurrences of the inattentional deafness phenomenon in each participant. We demonstrated that a task with high cognitive workload interfered with the ability to perceive an unpredictable auditory alarm. In parallel, auditory P300 amplitude diminished in the landing task with low load condition in comparison to the control condition and further diminished with an increase of the load (high load vs. low load). These results tend to confirm that our aviation landing task left few resources to process auditory alarm in the low-load condition and even fewer in the high-load condition. P300 amplitude could be used as a precursor to determine the susceptibility of an individual to alarm misdetection, even in absence of behavioral abnormalities.
